# Long-Term Recovery from Intimate Partner Violence: Recovery and Hope

**DOI:** 10.3390/ijerph192113825

**Published:** 2022-10-24

**Authors:** Mary Jean Carman, Frances Kay-Lambkin

**Affiliations:** 1 School of Medicine and Public Health, College of Medicine, Health and Well-Being, University of Newcastle, Newcastle, NSW 2308, Australia; 2 Hunter Medical Research Institute (HMRI), New Lambton Heights, NSW 2305, Australia

**Keywords:** intimate partner violence, recovery, hope, gender-based violence, healing

## Abstract

Recovery is a preferred outcome for assessing intervention effectiveness in the context of intimate partner violence (IPV), but measurement tools are in nascent form. It is therefore unclear what the recovery potential of survivors may be. A national online survey explored the self-rated recovery progress of Australian women (*n* = 1116), using visual analog scales (VAS) for recovery, hope, and other demographic variables. Findings show that many women rated themselves as completely recovered (14% of the eligible sample and 22% of the women who had left their partner > 10 years previously). However, most women experienced recovery as an ongoing process of healing (81%) and some women made little recovery progress (5%). Nevertheless, 77% of women who had separated >10 years ago rated their recovery as significant (scores of >70/100). Surprisingly, hope and recovery scores were only moderately correlated. This requires further investigation to determine what impacts on hope in long-term recovery, and how subjective and objective measures of hope and recovery vary in the context of IPV. The VAS was an efficient unidimensional measure for an online survey and is proposed for use in clinical and service contexts requiring subjective measures.

## 1. Introduction

It is widely accepted that intimate partner violence (IPV) is a global epidemic [1] and human rights violation [2]. Prevalence rates for an experience of IPV once in a lifetime in countries around the globe range from 10–53% [3] but “one in three” is the prevalence rate commonly cited, e.g., by the World Health Organization [4]. Additionally, one in three Australian women will experience IPV (including physical, sexual, and psychological forms of abuse) from a current or former dating or co-habiting partner [5].

The close relationship between developing empirically based definitions and determining prevalence rates in facilitating knowledge development and creating change has been an important lesson learned within the Australian [6] and global IPV sectors [7]. Definitions determine measurements and measurements determine what is known [8,9]. With accepted definitions and reliable measurement tools, researchers have been able to determine the nature and extent of IPV, including understanding the different types of abuse, the variety and extent of impacts of abuse, and the varied factors and social contexts in which abuse occurs [10,11]. Furthermore, having established the link between IPV and homelessness [12,13] and the intergenerational transmission of abuse [14,15] and in light of the lack of progress in overcoming IPV [16], there is now an emphasis on both prevention and intervention, to assist women and children affected by IPV [17,18,19].

The focus of intervention studies has recently turned to long-term recovery [20,21,22,23,24,25,26]. Women in long-term recovery have significantly different needs to their needs at the time of leaving; with pervasive health, mental health, financial, and social impacts manifesting over time [23,27,28,29]. Research attention has become firmly focused on determining how to measure the effectiveness of interventions to ascertain what is most helpful to women in supporting their recoveries [30]. Tools for evaluating intervention feasibility [31,32], and utilizing user-centered designs [33,34] are pertinent concerns. However, this work is only in its infancy [24,31] and knowledge development is hampered by the lack of relevant measurement tools and the lack of uniformity of outcome measures utilized across studies [34,35,36].

In Australia, recovery is a priority for researchers, but also policy writers, and service providers in the sector, and a general definition of recovery has been suggested in the draft of the fifth Australian research plan: “Recovery and healing refers to the ongoing process that enables victim survivors to be safe, healthy, and resilient, to have economic security, to recover and thrive” [37]. Although this is a good definition of recovery, there are no details of its empirical derivation, therefore it lacks the detail needed to operationalize into a recovery measurement scale. An empirically derived definition has been provided for use by the sector in developing tools to measure the recovery effect of interventions, including details of components that contribute to that definition, which can inform items in a scale—“For the women in the survey, recovery meant ensuring their safety and surviving independently of the perpetrator, while gaining freedom from his control; healing and moving on from the effects of the abuse, while enjoying a better life. Children’s well-being was of primary importance to their mothers and parenting responsibilities complicated their journeys to recovery” [38]. This definition is based on five themes emerging from the definitions of recovery provided by 665 Australian women who had separated from their abusive partner more than two years previously. These five themes referred to 22 dimensions integral to long-term recovery from IPV identified by the women, which could be used in the design of a multi-dimensional scale.

Common sense tells us that many women do recover from experiences of IPV, and many researchers believe in the possibility of recovery for survivors [21,22,23,39,40]. Even in the face of difficulty, “grief and relief are not mutually exclusive, survivors can reassemble a life informed by trauma that is no longer traumatic” [20]. However, there is no quantitative indication in the literature of what we can expect for survivors in terms of rates of recovery, time involved in recovery, and the extent of recovery possibilities. As Alexander [14] observed, “they [survivors] are after all, far from being a homogenous group in their perceptions of the violence they experienced, with stages of change unrelated to either recent or lifetime experience of violence.”

Further in the literature, there has been a subtle but persistent question over the recovery potential of all survivors, with various authors noting that some women seem to make little or no recovery progress [41,42,43,44,45,46]. Other researchers have qualified their expectations for women’s recovery, noting the length of time involved [47,48], the many factors involved, including factors on a social rather than an individual level [49], and the influence of other barriers to recovery, such as mental health impacts [29,48]. In one Australian study (*n* = 123), the author conceived of recovery as an ongoing experience, and noted the range of long-term impacts in the lives of survivors, claiming that “the evolving and expanding nature” of these impacts caused many participants to either criticize or consciously reject “recovery” terminology [43]. Evans [43] concluded her study by saying, “An abusive relationship will reverberate, in a variety of way and degrees, for the remainder of the lives of most survivors.” A concern is that, while Evans claims to have “bracketed out” her personal experience and beliefs (as a survivor who experienced ongoing contact with the perpetrator through shared child custody), her effectiveness in doing so cannot be assessed. As data collection occurred in focus groups, group effect or group bias may still have influenced her findings. Group bias (seeking to maintain consensus with the group) and people’s unwillingness to contradict group norms in face-to-face discussions can be problems in focus group research [50].

Adding to these concerns, the clinical guidelines for treating domestic and family violence (DFV), known as the White Book, which is provided by the Australian Royal Australian College of General Practitioners (RACGP) to their member doctors, has included a similar statement [51]. This book is a wonderful resource guiding doctors in assisting women who experience DFV (including IPV). However, when describing what happens to women after leaving an abusive relationship, the authors claim that: “Few will recover totally from the experience” [51]. This is a concerning statement as no evidence is cited to support this opinion and the statement may have significant ramifications for survivors. Doctors often act as the ongoing referral agents in the recovery process for survivors, and they need to hold proven beliefs about recovery possibilities for survivors to avoid the possibility of causing further harm.

Until now, there has been no accepted measure for recovery in the context of IPV, although research attention has turned to the task and a measure for “healing from gender-based violence (GBV-Heal)” has been offered by researchers in North America [52]. Construct validity for the GBV-Heal was developed from interviews (*n* = 56) and focus groups (*n* = 26) drawn from the individuals who had received care through an “academic medical centre” in one Michigan university [52]. This tool was then tested on a larger sample (*n* = 236) drawn from the same source. This was followed by a test–retest reliability study based on a new sample of 47/64 participants drawn from an organization, described as “a national health volunteer registry created by several academic institutions and supported by the U.S. National Institutes of Health” [52]. The GBV-Heal tool has included important dimensions of recovery—namely “trauma processing and self-advocacy, self-connection, relating to others, regaining hope and power” [52]. However, it does not include the following empirically derived dimensions of recovery—physical safety, financial independence and survival, stable housing, and parenting issues—which women continue to struggle with long after separation, particularly women on low-incomes [27,29,34,38,53]. For example, King and colleagues found financial issues to be a major and overlooked aspect of long-term recovery in their convenience sample of American women in long-term recovery (*n* = 130) [40]. Therefore, the GBV-Heal tool needs to be revised to improve its construct validity to ensure that it covers the relevant dimensions of recovery for more diverse populations [54] and to ensure that it is meaningful to those it seeks to measure [34,55].

While the concept of recovery has resonated with researchers, and momentum has quickly focused on recovery as an outcome, the important question is whether the concept of recovery also resonates with survivors [34]. A phenomenology of 23 Australian women conducted prior to this online survey by the authors (but unpublished), suggested that some women do not consider they have made significant recovery progress. It is important to resolve questions about the possibility of recovery for women, as both overstating and understating possibilities can be detrimental to survivors. Furthermore, recovery and hope are often associated in the mental health recovery literature [56], but it is not yet clear how, or if, they are associated concepts in the context of IPV. Some researchers in the IPV sector have also identified hope as important to long-term recovery [57,58], but more evidence is needed to determine whether hope is a component of recovery or an outcome of recovery. More research is also needed with samples not drawn from shelters, refuges, or universities [58,59].

The purpose of this study was to explore the self-rated recovery progress of women who had experienced IPV more than 2 years previously. Demographic details, mental health diagnoses (MHD), and service usage, along with two self-rated retrospective scale measures (recovery and hope) were used to consider the extent to which women felt recovered over the long term, and whether hope was associated with recovery as the recovery literature in the mental health sector suggests. The study aimed to answer the following research questions:

Is recovery from experiences of IPV possible?
How do women who have experienced IPV recover over the long-term period?
Is there an association between hope and recovery in the context of IPV?

Scale measurement tools were chosen for their efficiency of use in online surveys and for their suitability for use in psychometric studies [60], to explore the latent dimensions of a construct, or to measure attitudes, feelings, and behaviors in relation to a particular phenomenon [61]. Scale measurement tools can be either unidimensional or multi-dimensional in form. While multi-dimensional tools are highly valuable for in-depth assessments, the realities of clinical and service support limitations (in time, cost, and imposition on clients) limit their applicability [55]. Quick, easy-to-administer tools such as unidimensional scales are valuable as an adjunct to any multi-dimensional scales (MDS) available. The visual analog recovery scale (VAS) piloted in this study is one such unidimensional scale and is proposed as a subjective measure of progress in recovery from IPV. It is hoped that further discussion and exploration among researchers can occur, while a multi-dimensional scale is developed for use in tandem with this unidimensional scale. In summary, MDS is useful for the objective measurement of a construct but takes a lot of time and cost to develop and administer. Alternatively, the unidimensional scale is useful as a quick and easily administered subjective assessment of a construct, but care must be taken to appreciate the limitations of interpretations possible with this tool.

## 2. Methodology

This study comprised a national online survey examining the recovery experiences of women in Australia *(n* = 665) who had left an abusive intimate partner more than 2 years previously. Qualitative data from the survey provided an empirically derived definition of recovery [38]; the survey illustrated the enormous diversity of women’s experiences and made a case for larger sample sizes in the sector. Eligibility criteria included being a self-identified Australian woman, aged 18 or over and in long-term recovery (LTR) from IPV, defined as two or more years since abuse ended, to address a weakness in the literature. Women also needed sufficient English language and computer skills to complete the survey. Human research ethics approval was obtained from the University of Newcastle Human Research Ethics Committee (Approval no. H-2018-0037) before data collection began.

### 2.1. Sample

A total of 1119 people responded to the survey. The resultant survey sample included 1116 self-identified women from across Australia and three men whose responses were kept for a future survey addressing the needs of men in recovery from IPV. Of these women, 665 were eligible, as it had been more than 2 years since the abuse ended (the LTR sample). Despite the eligibility criteria, 451 ineligible women (40% of the total sample) completed the survey. For these women, it had not been more than two years since abuse ended and it is not clear whether these ineligible women did not read the survey information sheet, misinterpreted the eligibility criteria, or simply disregarded the eligibility criteria to participate. As there was no reward for participation, this was an unexpected outcome and created a dilemma over whether to retain the data. However, it was recognized that these contributions created a valuable opportunity as a comparison group for the eligible women (the LTR sample). Using the time since abuse ended (TSAE) variable in IBM SPSS Statistics Software 26.0, the total sample was split into two smaller samples: short-term recovery (STR) and long-term (LTR).

### 2.2. Measures

Survey questions covered demographic variables, variables related to MHD and services usage, and two retrospective self-rated scales—recovery and hope. The demographic details collected included gender, age, location, and ethnicity. Details of length of abuse (LOA) and TSAE were the only descriptors of abuse collected, as the required focus was on recovery experiences. Participants also nominated which mental health conditions they had been diagnosed with, from a list that included depression, anxiety, post-traumatic stress disorder (PTSD), substance abuse, and other. Participants were able to select more than one option in their answers. Another question elicited responses on the number and type of services women consulted during their recovery. All questions in the survey included a “prefer not to say” option (PNTS).

Recovery and hope were two purpose-built, retrospective, self-rated, continuous scales: visual analog scales (VAS) [62]. Each measure produced a continuous scale, indicated by a simple line with a sliding bar. The endpoints of the line were labeled “recovery journey just starting” (indicating unrecovered) to “recovery journey has ended” (indicating recovered). In this way, participants assessed their own progress in recovery using their subjective understandings of the concept. Similarly, in a second VAS, participants were asked to respond to the statement “My hope for the future is…” by sliding the bar from “hopeless” on the far left to “hopeful” on the far right.

Visual analog scales have been in use since 1921 [63] and can be described as a continuous line between two points labeled as the extremities of any nominated continuum, upon which a participant marks their subjective experience [63]. These scales are usually shown as an unmarked line of 10 cm long but numerous studies have proven that the actual length of the line does not change the validity of the VAS, as long as the features of the VAS are clearly visible and the endpoints of the scale can be accessed from all devices including smartphones [64]. Keeping the scale unmarked removes the problem of determining values represented by segments. VAS is scored by measuring from the left or start of the line to the point marked by the participant, giving a continuous scale scored from 1–10 or 1–100.

It is important to note that VAS scores are completely subjective and do not represent an absolute measure of the phenomenon. The score as used in this study has value as a “once-off” snapshot of a participant’s subjective state at a given point in time. If repeated measures were taken, then the score could be compared to the participant’s previous scores to assess any progress in relation to the phenomenon being assessed [64].

A strong case can be made for the use of VAS in measuring psychological phenomena, as many different subjectively assessed conditions have been reliably and validly measured using VAS, including quality of life, happiness, and stress [65]. Claims have been made that VAS can achieve validity and reliability scores equal to or above similar MDS for many health, mental health, and social outcomes and this appears to apply to the psychometric assessment of complex as well as simple conditions [65]. According to Byrom et al. [64], VAS are especially suited to patient ranking of outcome measures, assessing a single construct with many perceptible gradations, and showing sensitivity to changes. These scales can be used with parametric statistics, in contrast to Likert scales, which have discrete intervals and need non-parametric statistical analysis [66].

The recovery VAS in this study was used to triangulate with the qualitative data collected in the survey. It is not offered as a validated and reliable measure but instead provides a snapshot of a large sample at one time and place. Without a proven valid and reliable multi-dimensional scale to use to validate the VAS, it is proposed as a “fit for purpose” tool and a catalyst for further studies. Test–retest reliability and sensitivity of the VAS as a recovery measure tool needs to be established and researchers are encouraged to explore this issue. Using the recovery VAS should not negate the importance of well-built MDS, but a VAS could augment MDS and become an important tool in the clinician/interventionist’s toolbox. There is a need for efficient low-cost methods of assessing recovery in the population of women who have experienced IPV, when a subjective view is needed and time, cost, and convenience do not support the use of MDS if one were available.

### 2.3. Recruitment

A paid Facebook social media campaign targeted self-identified women, Australia-wide, who were over 18 years of age and had experienced IPV more than two years previously. The campaign ran for 22 weeks, according to the following 4-week cycle: AUD 100 per day for 7 days, then 14 days with no advertising, then a final 7 days of advertising at AUD 100 per day. Several short-burst campaigns, as recommended by Facebook, also ran concurrently. Facebook advertisements invited women to click on a link that took them directly to the online participant information statement and consent process for the study. Once this was completed online, women clicked on a final link to complete the online survey using the SenseMaker platform. The survey took approximately 30 min to complete. No incentive other than intrinsic reward was offered to respondents.

### 2.4. Data Analysis

It is important to note that the study was exploratory, not explanatory, and concerned with descriptive statistics and observations rather than with hypothesis testing. Only statistically significant findings (*p* < 0.05) are reported where they help to describe women’s recovery experiences. The survey data were de-identified and then imported into SPSS v 26.0 for analysis. Sample characteristics were explored using frequencies and percentages or means and standard deviations, as appropriate. Chi-squared tests explored any association between categorical variables and one-way analysis of variance (ANOVAs) explored any association between continuous and categorical variables. Two-tailed Pearson correlations explored any associations between continuous variables, with *p* < 0.05 indicating significance in the reported results. PNTS responses were coded as missing in SPSS.

## 3. Results

### 3.1. Demographics

Table 1, below, includes the demographic details for the participants, divided into LTR (eligible sample), STR, and the total sample.

#### 3.1.1. Age

Participants in the survey ranged in age from 18 years to more than 65 years. Overall, there were fewer younger women than older women in the total sample, and this was consistent across the LTR and STR samples. (χ^2^ (*df* = 4, *n* = 1097) = 44.244, *p* < 0.001). Noticeably fewer women in the 18–25 years age bracket participated in the study.

Within the LTR sample, chi-squared analyses identified statistically significant associations between age and ethnicity (χ^2^ (2, *n* = 650) = 10.778, *p* = 0.005), age and anxiety (χ^2^ (1, *n* = 664) = 20.147, *p* < 0.001), and age and any MHD (χ^2^ (1, *n* = 664) = 5.591, *p* < 0.018). In relation to age and ethnicity, women aged 46 years and over were statistically more likely to have arrived in Australia from another country or to identify as being of Aboriginal and/or Torres Strait Islander (ATSI) background than were younger women. In relation to age and anxiety, women aged 35 years and younger were statistically more likely to have a diagnosis of an anxiety disorder or any MHD, than were women older than 35 years.

#### 3.1.2. Location

Due to the small sample size of remote women, rural and remote women were merged into one category comprising 27% of the LTR or eligible sample; this was a strength of the sample. Similar proportions of women were observed across locations within the STR and LTR samples.

#### 3.1.3. Ethnicity

Most women in the study identified as being born in Australia. In the LTR sample, 80% of women identified as being born in Australia, as did 83% in the STR sample. A further 3% of women in both the STR and the LTR samples identified as ATSI. Women who identified as “Arrived in Australia” formed 15% of the LTR sample and 13% of women in the STR group.

#### 3.1.4. Length of Abuse (LOA)

Of the total sample, only 10% had experienced a period of abuse of fewer than 2 years. Of all the women who responded to the survey, 90% had endured abuse for periods longer than 2 years, and 47% of these women had endured abuse for more than 10 years. As might be expected, women aged 35 years and over were more likely than women aged 35 and under to have experienced abuse for more than 10 years (χ^2^ (2, *n* = 1112) = 95.196, *p* < 0.001). However, it is important to note that women aged 18–25 years in this sample still reported experiencing abuse for long periods, with 42% of these women reporting abuse for more than 2 years and one woman for 5–10 years.

LOA in the LTR sample was significantly associated with age (χ^2^ (2, *n* = 663) = 51.669, *p* < 0.001), PTSD (χ^2^ (2, *n* = 664) = 9.355, *p* ≤ 0.009), employment service usage (χ^2^ (2, *n* = 664) = 12.804, *p* ≤ 0.002), and service usage count (χ^2^ (10, *n* = 664) = 19.351, *p* ≤ 0.036). Women who had experienced abuse for 10 years or more were significantly more likely to be 35 years or older, have a diagnosis of PTSD, and have consulted employment services and two or more other services than were women who had experienced abuse for 10 years or less.

#### 3.1.5. Time since Abuse Ended

Using the total sample, a chi-squared test of independence between the categorical variables of age groups (under and over 35 years) and TSAE indicated there was a statistically significant association between the length of TSAE and the two age groups (χ^2^ (4, *n* = 1097) = 106.811, *p* < 0.001). Women aged 35 and over were more likely to be in LTR than women under 35 years (χ^2^ (4, *n* = 1097) = 106.811, *p* < 0.001). However, 10% of older women (65+ years) reported abuse less than 6 months previously. No other statistically significant associations were found between TSAE and any other demographic variable.

#### 3.1.6. Mental Health Diagnoses (MHD)

The variable MHD was split into three groups: 0 × MHD, one–two × MHD, and three or more MHD. Of the women in the LTR sample, only 19% did not have an MHD. Of the 81% of women in the LTR sample who had an MHD, 51% reported one or two diagnoses and 30% reported more than three to five diagnoses. Of the 83% of women in the STR sample who reported having an MHD, 49% reported having one or two diagnoses and 34% reported having more than two MHD.

For the LTR sample, anxiety, PTSD, and depression were similarly common, with each one being present in approximately half the women (50%, 49%, and 48%, respectively), indicating a high rate of comorbidity of mental health issues in these women. For the STR sample, anxiety, PTSD, and depression were slightly more common, with the prevalence of each (55%, 51%, and 50%, respectively), indicating a higher rate of PTSD and comorbidity of mental health issues than in the LTR sample, as might be expected. Substance-use disorders were less prevalent than anxiety, PTSD, and depression, impacting only 12% of the LTR and STR samples.

MHD were statistically significantly higher in LTR women with substance-use disorders as 85% of these women reported three or more diagnoses (χ^2^ (*df* = 2, *n* = 665) = 135.130, *p* < 0.001). In the STR sample, MHD were also statistically significantly greater in women with substance-use disorders; 89% of these women reported three or more MHD (χ^2^ (*df* = 2, *n* = 451) = 85.422, *p* < 0.001).

#### 3.1.7. Services Consulted

According to the count of services used, most women in the LTR sample had consulted at least one service in their recovery, with only 10% of women reporting they did not consult any service at all. More than half the LTR sample (67%) consulted one to two types of service, and approximately a quarter (24%) of women consulted more than two types of service. In the STR sample, slightly more women (70%) had consulted one to two services in their recovery, and only 8% had not consulted any service at all.

The LTR and STR samples varied slightly from each other in the types of services that the women consulted. Women in the STR sample compared with women in the LTR sample used more healthcare (69% vs. 66%) and other services (28% vs. 25%), respectively. Fewer women in the STR sample than in the LTR group consulted employment services and housing services. Use of charities was about the same across the two samples.

#### 3.1.8. Scale Measures: Recovery Scale

The self-rated recovery scale represented the extent to which participants felt recovered from their experiences of IPV on a VAS from 0 (recovery journey just starting) to 100 (recovery journey ended). Table 2 displays the mean recovery scores of participants for the different samples.

*T*-tests compared the recovery scores for women according to the TSAE (2 years or less vs. more than 2 years). Results indicated that the mean recovery score in the LTR sample (72.58) was statistically significantly higher than the mean recovery score of the STR sample (50.71). The statistical significance of the association between recovery scores and the two samples was (*t* = 14.497, *df* = 1106; *p* < 0.001). Considering only the women who identified as more than 10 years since abuse ended, the mean recovery score was 78.95.

**Figure 1:**
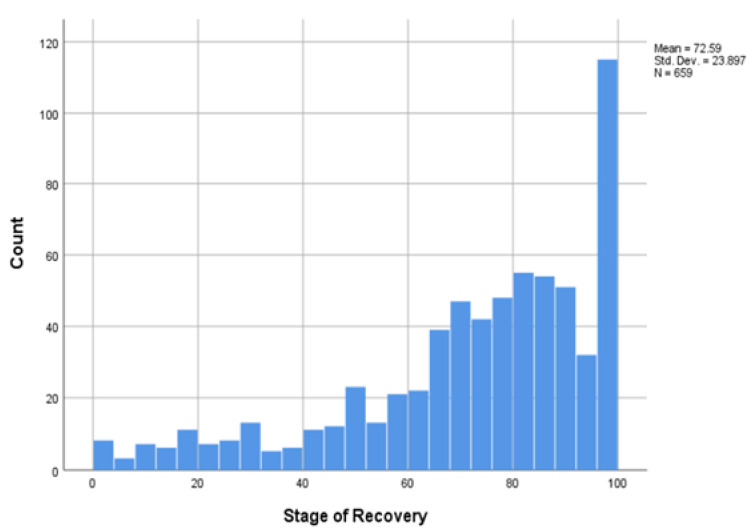
Distribution of self-rated recovery scores (visual analog scale 0–100) for women in long-term recovery from intimate partner violence (more than two years since abuse ended).

The number of women who rated themselves as recovered by selecting a VAS of 98 or more was considerably higher in the LTR sample (13.8% of women), than in the STR sample (1.33%). This illustrates the possibility of LTR for many women, although not all women do recover. Importantly, 5.3% of women in the LTR sample selected a VAS of below 20 (raw score of 0–19.99), indicating that they had made very little recovery progress, despite the long TSAE. The percentage of women in the STR sample (16.5%) choosing a VAS of below 20 was approximately three times higher than in the LTR sample, illustrating that as time passes many women will experience some progress in their recoveries. As indicated in Figure 1 below, many women (14%) whose abuse ended 2 or more years earlier considered themselves to be completely recovered from partner abuse by nominating a VAS of 98/100 or higher. If we look just at the women whose TSAE is more than 10 years in Table 2 above, 22% rated themselves as fully recovered, and this equates to 1 in 5 women. However, for most women in the LTR sample (81%), recovery is ongoing, and a small proportion of women (5%) struggled to make significant recovery progress even many years after leaving an abusive relationship. Moreover, 77% of women whose TSAE was more than 10 years ago in Table 2 above, rated their recovery as significant, by nominating a VAS of 70/100, or more.

The strength of the statistically significant association between recovery and TSAE according to Pearson’s chi-squared test was (χ^2^ (*df* = 4, *n* = 659) = 28.783, *p* < 0.001).

#### 3.1.9. Scale Measures: Hope Scale

This scale represented the extent to which the participants felt hopeful about their recovery from partner abuse, with participants self-rating their level of hope on a scale of 0 (hopeless) to 100 (hopeful). Table 3 displays the mean VAS hope scores of the women in the different samples as a function of time since their abuse ended.

Women who had left the abusive relationship 2 or more years earlier (27.67%) indicated they were the most hopeful they could feel, by scoring themselves 98 or above on the VAS for hope. Only 10% of the LTR sample scored themselves 50 or below on the scale, and only 14 women (2.11%) ranked themselves under 20 on the scale, indicating their lack of hope, or their hopelessness. Possible reasons for the women’s lack of hope were considered and may have included their locality and mental health status. These women were more likely to come from remote (two women), rural (five women), and regional localities (six women); no urban women were found in this group. These women were also more likely to have multiple MHD; only one woman had no diagnosis, four women had 1 or 2 MHD, eight women had three or more MHD and one woman did not disclose her mental health status. Of the diagnoses, 12 women had depression, nine had anxiety, eight had PTSD and four had substance-use disorders, again illustrating a high level of comorbidity.

**Figure 2:**
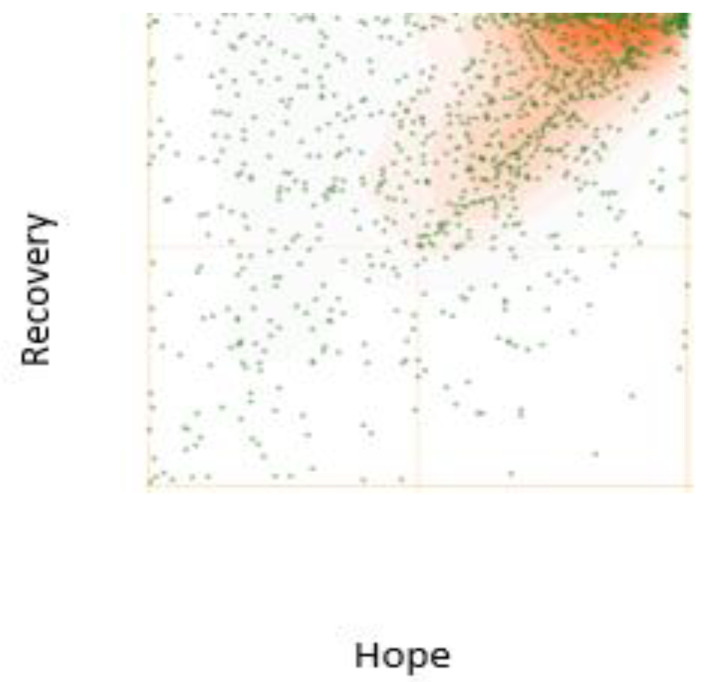
Distribution of self-rated hope scores (visual analog scale 0–100) for women in long-term recovery from intimate partner violence (more than 2 years since abuse ended).

Figure 2 indicates that most women ranked themselves as hopeful in relation to their recovery from partner abuse. Levels of hope were considerably higher in the LTR group (27.7% ranked >98/100); than for women who had more recently left their abusive partners (13.4% ranked >98/100)—see Table 3 above.

Using the LTR sample, a summary of the significance of the ANOVAs undertaken in this study is presented in Table 4 [67], along with the relevant *p*-value for each ANOVA. Where there are statistically significant differences, post hoc analyses identified where the differences occurred. Tests of normality were used as precautionary measures and are not reported with the ANOVA findings.

### 3.2. Recovery and Hope

#### 3.2.1. Recovery × Age (Six Groups)

A one-way between-groups analysis of variance (ANOVA) explored the impact of age groups on levels of recovery as measured by the self-rated recovery scale. There was a statistically significant difference at the *p* ≥ 0.05 level (95% confidence interval) in scores between the six age groups (*F* (5, 652) = 6.578, *p* < 0.001). Post hoc analysis revealed that significant differences occurred between the 66 + age group and the 26–35, 36–45, and 46–55 age groups (with group means of 24, 17, and 18, respectively), and between the 56–65 age group and the 26–35 age group (mean of 12).

#### 3.2.2. Recovery × Number of MHD

A statistically significant relationship existed between the number of MHD and recovery scores (*F* (2, 658) = 22.827, *p* < 0.001). Post hoc analysis revealed that these differences existed between 0 MHD and 3–5 MHD, as well as between 1–2 MHD and 3–5 MHD, indicating that recovery scores were statistically significantly lower for women with 3–5 MHD than for women with fewer diagnoses.

#### 3.2.3. Recovery × TSAE

TSAE was categorized as: “Less than 5 years ago”, “Less than 10 years ago” and “A long time ago” (or more than 10 years ago). There was a statistically significant difference in recovery scores between the three TSAE groups (*F* (2, 656) = 19.085, *p* < 0.001). Post hoc analysis revealed that this difference existed between those whose abuse ended less than 5 years ago and those women whose abuse ended “a long time ago” (*p* < 0.001).

#### 3.2.4. Recovery × LOA

LOA was categorized as “0–10 years” and “more than 10 years” of abuse endured by women. There was a statistically significant difference in recovery scores between the two LOA groups (*F* (1, 657) = 4.353, *p* = 0.037). Women who experienced abuse for longer than 10 years reported significantly lower recovery scores than women who endured abuse for 10 years or less (see Table 4).

#### 3.2.5. Hope × Locality (4 Groups)

A statistically significant difference in hope scores existed between the four localities represented in the survey (*F* (3, 652) = 2.749, *p* = 0.042), as shown in Table 4. Remote women reported significantly less hope than women in other localities.

#### 3.2.6. Hope × MHD

A statistically significant difference in hope scores emerged among the six MHD groups (*F* (5, 657) = 7.934, *p* < 0.001). In general, women with an MHD reported lower levels of hopefulness than women without an MHD. Post hoc analysis revealed statistically significant differences in ratings of hope for women with one, two, or three MHD versus those with five diagnoses, women with one diagnosis versus those with four diagnoses, and those with one, three, four, or five diagnoses versus those with no MHD. No significant differences existed between women with no MHD and women with two diagnoses.

**Figure 3:**
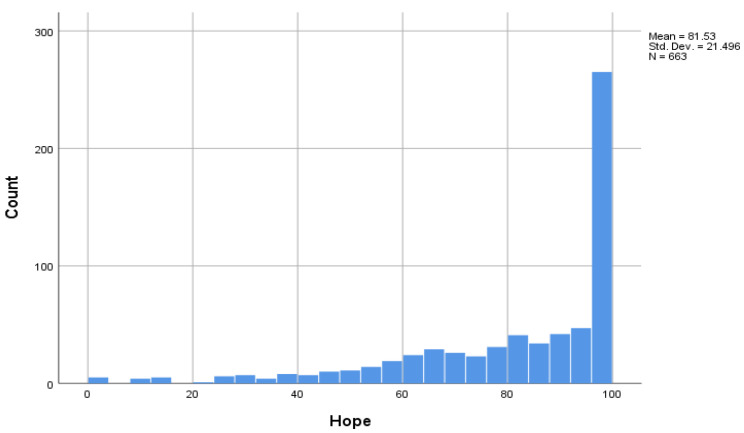
Self-rated recovery and hope scatterplot.

Correlational analysis using Pearson’s product–moment coefficient explored the strength and direction of the correlation between the recovery and hope scales. There was a moderate positive correlation between the self-rated recovery and self-rated hope scores (*p* = 0.541, *n* = 659, *p* < 0.001). The scatter plot illustrating this correlation is displayed in Figure 3 below.

## 4. Discussion

This study set out to examine the LTR of women who had experienced abuse from an intimate partner. Overall, women reported that recovery was something they identified with and experienced, and many expressed hopes about the future following the end of the abusive relationship. Implications of the findings are explored below.

It is not surprising that our data indicated younger women (35 years and under) were more likely than older women to have been in an abusive relationship within the last 10 years, as the prevalence of abuse has recently been confirmed to be highest in this age group [68]. Commensurate with this observation was our finding that perceptions of recovery generally increased with the increasing age of women in our sample and recovery generally progresses over time for most women. However, it is not yet known whether targeted recovery-oriented services can be effective in assisting women in their recoveries.

Recovery-oriented services aimed at the needs of different age groups will be important and services for younger women may need to be distinct in style and nature from those services provided for older women. Future studies could explore what aspects of service provision pertain to the differing needs of women from different age groups. Furthermore, although older women were more likely to be in LTR than younger women, some older women—including those over the age of 65—were experiencing ongoing abuse, and still required crisis support and help in leaving.

Cultural and linguistically diverse (CALD) women were significantly under-represented in the survey. Not only do recovery-oriented services need to consider the accessibility of their services to the diverse needs of these women, but more work remains in developing an understanding of how differently these services need to be structured, resourced, and delivered to meet the cultural needs of all people is needed. The provision of translation services is essential to ensure research participation for many CALD women.

We regard it as important that two-thirds of our sample (65%) were women from regional, remote, and rural areas of Australia, as prevalence rates are higher in these areas [69]. This supports the notion that recovery support services should be provided in regional centers and highlights the need for innovative service provision for women recovering from IPV in rural and remote areas, including the “Hub and Spoke” model [70]. This may become increasingly important as women may be drawn to the cheaper housing and accommodation found in these areas. Online service provision could be developed to help overcome distance barriers and assist with protecting the confidentiality of women in these areas. Utilizing general practice medical services (where they exist) to provide recovery services may also be helpful, as women attending medical services are less likely to be socially stigmatized in small rural communities and many of the women surveyed (2/3) sought assistance from health-related services.

This reinforces the importance of considering the general practice medical center as screening and referral agents in the LTR process for women and the feasibility of general medical practices being able to provide some form of support services for women in recovery remains to be investigated [34]. Mental health services and other allied health professionals also need professional development including assessment techniques for IPV, knowledge of IPV, and knowledge of the recovery support needs of women [34]. More research is needed in each of these areas.

It is a serious concern that approximately 90% of the women in our LTR sample reported enduring abuse for more than 2 years. Of these women, half had experienced abuse for up to 10 years and half had endured abuse for more than 10 years. This finding supports Alsaker and colleagues’ study [71], which found, in a sample study of 22 women in Norway, that the mean time for staying in an abusive relationship was 11 years. In our study, women who experienced abuse for longer than 10 years reported significantly lower perceptions of recovery than women who experienced abuse for shorter periods. In a related finding, Bonomi and colleagues’ study of 3429 American women, drawn at random from a large health plan, found longer duration of IPV was associated with incrementally worse health [72]. Herman [41] also reports that chronic or repeated experiences of trauma complicate and prolong the recovery of survivors. Further studies investigating the impacts of LOA on recovery potential may be helpful in determining the importance of addressing this variable in relation to women’s recovery.

Many of the women in our survey who were in recovery from IPV had mental health symptoms and one or more MHD. Compared to the national prevalence rate of 45% for any mental illness in a lifetime [73], the women in our study reported 1.8 times the national prevalence for MHD. Approximately a third of the women had three or more MHD and experienced significant difficulty in recovering from their experiences, yet women are not routinely screened by mental health services for their exposure to IPV [34,74]. The low rate of resolution of mental health issues over time may reflect the number of women unable to access support, or the entrenched nature of their injuries and other issues. Further research into the resolution of mental health issues (for clinical and sub-clinical level symptoms) over the LTR period is needed.

### 4.1. Recovery

This study has provided further insights into how women view their recovery from the impacts of IPV. Importantly, some women (14% of the LTR sample and 1% of the STR sample) reported that they fully recovered following their experiences of IPV (VAS of 98 or above). This rose to 22%, or one in five women, more than 10 years TSAE, who ranked themselves as fully recovered. For a general guide, we can say that 77%, or three out of four women, who were more than ten years since separation, rated their recovery progress as significant, with a score > 70/100.

In contrast, some women (5.3% of the LTR sample and 16.2% of the STR sample) ranked themselves below 20 on the recovery scale, indicating that they had made very little recovery progress. Again, looking at only the women with 10+ yrs TSAE, this rate drops only marginally to 4.1% and indicates a group of women who have “stalled” in their recoveries. Among these 35 women, all but 1 woman had at least one MHD and 63% of the women had more than one MHD. In all other aspects, the women appeared to be similar to the other samples. Clearly, women with multiple MHD find it more difficult to recover than women without an MHD, and more research is needed to understand the recovery trajectories of these women. It is important that the mental health sector take up the challenge to provide recovery services adapted to the unique needs of women in recovery from IPV, as research has shown that women experiencing both IPV and mental health issues often “fall into gaps in services” between the two sectors [75].

The time, cost, and ease of use of unidimensional measures such as the VAS when seeking to determine progress in recovery from the survivor’s perspective, may be all that is needed in a clinical or intervention setting, particularly when the assessment needs to be a quick and subjective one. VAS are easy to use and for this reason, achieve high response rates and levels of completion [76]. VAS are also more sensitive measures than MDS as they allow for a greater influence on mood and subjectivity. Further research is needed to explore the sensitivity and reliability of the VAS recovery scale.

The recovery VAS may be used in intervention settings in several ways. Most importantly, it should be utilized at the start of an intervention as a baseline measure of a survivor’s subjective assessment of their current state of recovery progress. Then, the measure may be used again (being careful to apply the measure in the same way) at the completion of the intervention, or at another nominated time (for example, 3 months later, 6 months later, and 12 months later). Differences in the scale measure over time will illustrate changes in a survivor’s appraisal of their recovery progress and thus whether that recovery progress has been impacted by the intervention. It may also indicate whether the impact was lasting. As the VAS is so quick and easy to use it can be used to map the recovery progress of individuals over time. Drops or increases in the VAS scores can be used by case managers to interrogate the possible factors impacting a client’s experiences. This can lead to enhanced understanding and service support initiatives for that client.

Finally, it is important for interventionists to remember that the recovery VAS is a subjective (or personal) assessment of recovery progress and not an objective measure (independent assessment). This means that it is valid to compare a person’s recovery progress using their own measures over time, but it is not valid to compare the scores between different people as an objective measurement tool is needed for that. Differentiation between the subjective experience of recovery and the objective assessment of recovery factors as defined in a multi-dimensional scale is a necessary concept for interventionists to consider. It is important to acknowledge the differences between these two distinct constructs. For example, a 22-year-old woman may rate herself as “fully recovered” (subjective assessment) because she had successfully left the abusive relationship, been successful in her employment achievements, overcome her addictions and mental health issues, and had established a large and supportive social network in a different city. She was also happy and fulfilled in her life. However, an objective observer may notice her experiences of persistent trauma symptomology which may impact the assessment of her recovery status if an objective multi-dimensional tool was used for the assessment. That is, assuming that the impact of ongoing trauma symptomology was included as a component in any multi-dimensional measure of recovery from IPV (objective assessment), and it is our opinion that it should be. Interventionists may wish to explore the roles of these two distinct constructs of recovery, and their influence on the recovery context, once a multi-dimensional scale is available.

### 4.2. Correlations

The results of this study indicate that subjective assessments of hope are associated with recovery from IPV [58], but the correlation is only in the moderate range. This suggests that hope might not be as integral to recovery from IPV as the mental health recovery literature appears to indicate [56,77]. As indicated in Figure 3 above, recovery may come first, as indicated by the number of women in the top left quadrant, with high recovery scores but low hope scores. Then, as recovery progresses, hope may rise, and the momentum continues to increase. In this case, hope is not necessarily a driver of recovery as suggested in the literature [78], nor even an element of recovery, but may instead be a by-product or an outcome of recovery. Results from the qualitative data collected in the same survey provide some insight into the link between recovery and hope. It does appear that recovery progress produces hope which in turn produces recovery progress. This hypothesis needs to be tested in further studies.

Further, the difference between subjective unidimensional measures and more objective multi-dimensional measures needs to be established before comparisons are made between these differing measures.

### 4.3. Limitations of the Study

The online, anonymous, Australia-wide survey and large sample size were definite strengths of the study. Only retrospective self-reported measures were possible and recall biases may have impacted the findings. In accordance with the exploratory nature of the study and the broad sweep of findings, measures indicated broad trends only and were not intended to be prescriptive. As this is a cross-sectional study, it is not possible to infer causation from these results. For example, hope and recovery are associated but it is not clear whether hope drives recovery or recovery drives hope and what mediating and moderating factors impact the association between them.

Other limitations include the fact that the survey sample was biased by including only women who had access to Facebook and associated social media and who self-selected to be included in the study. The sample was also slightly biased against younger women. This may have been because Facebook is no longer the preferred social media outlet for younger people, or because the visual image used to promote the study was of an older woman. The latter point is at least partly true, as a change to a younger image later in the campaign produced an increase in younger participants undertaking the survey.

Future studies are needed to investigate LTR from IPV for women in countries around the world, for men, and for people of CALD backgrounds. Government funding for translation services would help to reduce barriers to participation in the future. Gaining accurate assessments of the participation of women who do not want to reveal personal characteristics of gender and ethnicity may continue to be problematic for researchers. Future research exploring the relationship between hope and recovery is also needed.

Finally, difficulties interpreting the questions or manipulating the scales may also have influenced responses. The design of the scale was chosen to minimize difficulties in answering closed-ended questions such as “Do you feel you have recovered from your experiences of IPV?” The removal of numerical values from the scale was also intentional, to mitigate the problem of assigning a specific value (and the problem of determining the meaning assigned to that value), rather than indicating a general relative position. The use of a VAS for recovery needs validation when we have a multi-dimensional scale with adequate construct validity. Other researchers could explore the test–retest reliability of the tool, as ethics approval to retain the identity and contact details of respondents was not granted for this survey, due to confidentiality concerns.

## 5. Conclusions

Many women rate themselves as fully recovered from their experiences of IPV, illustrating that recovery from partner abuse is possible and resonates as a concept for women impacted by IPV. However, some women made little or no recovery progress, even after many years, and the experiences of these women need to be explored. Mental health issues were influential on their recovery potential. Further, as time passing is associated with recovery, most women will experience recovery as an ongoing journey.

Hope and recovery are associated, but the association is only moderate. Our findings question whether hope inspires recovery as theorized, or whether recovery inspired hope. Further research is needed to test the association between the two variables.

There is a gap in the literature about the impacts of LOA on women’s health, quality of life, and recovery progress. Given the long periods of time over which many women endure abuse, further research is needed to explore the hypothesis that LOA intensifies adverse impacts from IPV and delays recovery.

## Figures and Tables

**Table 1:** Demographic data for all samples.

Variable	Long-Term Recovery (LTR)			Short-Term Recovery (STR)			Total Sample	
	(*n* = 665)			(*n* = 451)			(*n* = 1116)	
	ƒ	% LTR	% Total	ƒ	% STR	% Total	ƒ	%
TSAE:								
PNTS:				18	4	2	18	2
<6 months ago				203	45	18	203	18
<2 years ago				230	51	21	230	21
<5 years ago	227	34	20				227	20
<10 years ago	193	29	17				193	17
A long time ago	245	37	22				245	22
	665	100	60	451	100	40	1116	100
Gender:								
Female	661	99		448	99		1109	99
Other Genders	4	1		3	1		7	1
	665	100		451	100		1116	100
Age:								
18–25	7	1	1	12	3	1	19	2
26–35	83	12	7	90	20	8	173	16
36–45	180	27	16	162	36	15	342	31
46–55	220	33	20	131	29	12	351	32
55–65	143	22	13	46	10	4	189	17
65 plus	31	5	3	9	2	1	40	4
	664	100	60	450	100	40	1114	98
Location:								
Remote	7	1	1	8	2	1	15	1
Rural	175	26	16	117	26	10	292	26
Regional	252	38	23	179	40	16	431	39
Urban	220	33	20	138	31	12	358	32
	654	98	59	442	98	40	1096	98
Ethnicity:								
Aboriginal/Torres Strait Islander	20	3	2	13	3	1	33	3
Arrived in Aust.	100	15	9	57	13	5	157	14
Born in Aust.	531	80	48	374	83	34	905	81
Other	13	2	1	5	1	0	18	2
	664	100	59	449	100	40	1113	100
Length of Abuse:								
Once	2	0	0	2	0	0	4	0
Up to 6 months	6	1	1	5	1	0	11	1
6 months–2 years	60	9	5	39	9	3	99	9
2 up to 5 years	139	21	12	81	18	7	220	20
5 up to 10 years	158	24	14	102	23	9	260	23
More than 10 years	299	45	27	221	49	20	520	47
	664	100	59	450	100	40	1114	100
Mental Health Diagnoses:								
Anxiety	331	50	30	249	55	22	580	52
PTSD	329	49	29	232	51	21	561	50
Depression	317	48	28	227	50	20	544	49
SUDs	83	12	7	55	12	5	138	12
Other	61	9	5	54	12	5	115	10
MH Count 3 groups								
0	126	19	11	75	17	7	201	18
1–2 diagnoses	340	51	30	223	49	20	563	50
3–5 diagnoses	199	30	18	153	34	14	352	32
	665	100	60	451	100	40	1116	100
Any MH Diagnosis:								
No	126	19	11.29	75	17	7	201	18
Yes	539	81	48.30	376	83	34	915	82
	665	100	60	451	100	40	1116	100
Services Used:								
Employment	179	27	16	110	24	10	289	26
Housing	181	27	16	104	23	9	285	26
Healthcare	439	66	39	312	69	28	751	67
Charities and churches	170	26	15	111	25	10	281	25
Other	166	25	15	128	28	11	294	26
Service Count 3 groups:								
None	66	10		36	8		102	9
1–2	442	67		315	70		757	68
3–5	157	24		100	22		257	23
	665	100		415	100		1116	100
Recovery Measure:								
0–19.99 Not recovered	35	5.31		73	16.30		108	9.75
20–97.99 Recovering	533	80.88		370	82.40		903	81.50
98–100 Recovered	91	13.8		6	1.30		97	8.75
	659	100		449	100		1108	100
Hope Measure:								
0–19.99 Hopeless	14	2.10		28	6.20		42	3.80
20–97.99 Hoping	465	70.10		348	80.40		826	74.30
98–100 Hopeful	184	27.80		73	13.40		244	21.90
	663	100		449	100		1112	100

Note: TSAE: time since abuse ended; PNTS: prefer not to say; MH: mental health; SUD: substance-use disorders.

**Table 2:** Self-rated recovery VAS scores for women according to length of time since abuse ended.

STR Sample			2–5 Years TSAE	5–10 Years TSAE	10 Years + TSAE	LTR Sample	Total Sample
*n*	Valid	449	226	189	243	659	1108
	Missing	2	1	4	2	6	8
Mean		50.71	65.62	72.76	78.95	72.58	63.72
Median		55.16	70.28	78.89	84.28	77.80	69.09
Std Deviation		25.73	24.76	22.96	22.05	23.89	26.88
Bottom 10% of scale scores		<14.72	<24.69	<40.68	<49.22	<33.81	<20.49
Top 10% of scale scores		>81.25	>92.19	>98.80	>99.43	>98.83	>97.24
Percentage of women scoring > 90			12.80%	24.30%	39.90%		
Percentage of women scoring > 70			50.00%	63.50%	77.50%		

Note: LTR = long-term recovery; TSAE = time since abuse ended; VAS = visual analog scales.

**Table 3:** Self-rated hope visual analog scores for women according to length of time since abuse ended.

		LTR Sample	STR Sample	Total Sample
*n*	Valid	663	449	1112
	Missing	2	2	4
Mean		81.52	69.10	76.51
Median		90.29	73.60	83.51
Bottom 10% of scale		<50.2	<29.1	<20.5
Top 10% of scale		>99.4	>99.1	>97.2

Note: LTR = long-term recovery; STR = short-term recovery.

**Table 4:** Summary of *p*-values for one-way ANOVA and post hoc analysis where relevant.

Age and Recovery				Locality and Hope			
*p* < 0.001				*p* = 0.042			
Age Groups (All 6 groups)	*n*	Mean	SD	Locality	*n*	Mean	SD
18–25	7	68.79	22.83	Remote	7	62.52	43.79
26–35	82	65.01	26.13	Rural	174	83.09	20.51
36–45	177	71.75	24.08	Regional	252	80.20	22.05
46–55	219	70.52	23.85	Urban	219	82.73	19.74
56–65	143	77.88	21.23	Total	652	81.63	21.29
65 plus	30	89.08	17.70				
Total	658						
**Number of Mental Health Diagnoses (MHD) and Recovery**				**Number of Mental Health Diagnoses (MHD) and Hope**			
***p*** ** < 0.001**				***p*** ** < 0.001**			
**No. of Diagnoses**	***n***	**Mean**	**SD**	**No. of Diagnoses**	***n***	**Mean**	**SD**
0	125	80.56	19.13	0	91	90.22	13.6
1–2 MHD	338	74.70	22.59	1	276	82.47	20.69
3–5 MHD	196	63.84	26.20	2	99	81.35	21.64
Total	659	72.58	23.89	3	120	79.18	23.32
				4	68	73.67	23.24
				5	9	56.95	29.92
				Total	663	81.52	21.49
**Time Since Abuse Ended (TSAE) Groups and Recovery**				**Length of Abuse (LOA) and Recovery**			
***p*** ** < 0.001**				***p*** ** = 0.037**			
**TSAE Groups**	***n***	**Mean**	**SD**	**LOA**	***n***	**Mean**	**SD**
Less than 5 years ago	226	65.63	24.77	0–10 years	362	74.32	22.99
Less than 10 years ago	189	72.76	22.96	More than 10 years	296	70.42	24.85
A long time ago	244	78.89	22.02	Total	658	72.57	23.91
Total	659	72.58	23.89				

## Data Availability

Requests for access to the data should be directed to the corresponding author. The data are not publicly available as this was a condition of ethical approval to protect participants privacy and confidentiality.
